# CyProQuant-PCR: a real time RT-PCR technique for profiling human cytokines, based on external RNA standards, readily automatable for clinical use

**DOI:** 10.1186/1471-2172-6-5

**Published:** 2005-03-04

**Authors:** Philippe Boeuf, Inès Vigan-Womas, Delphine Jublot, Séverine Loizon, Jean-Christophe Barale, Bartholomew Dicky Akanmori, Odile Mercereau-Puijalon, Charlotte Behr

**Affiliations:** 1 Unité d'Immunologie Moléculaire des Parasites, URA CNRS 2581, Département de Parasitologie et de Médecine Moléculaire, Institut Pasteur, 28 rue du Dr. Roux, 75724 Paris Cedex 15, France; 2 Unité d'Immunophysiopathologie Infectieuse, URA CNRS 1961, Département d'Immunologie, Institut Pasteur, 28 rue du Dr. Roux, 75724 Paris Cedex 15, France; 3 Immunology Unit, Noguchi Memorial Institute for Medical Research, PO box LG 581, Legon, Ghana

## Abstract

**Background:**

Real-time PCR is becoming a common tool for detecting and quantifying expression profiling of selected genes. Cytokines mRNA quantification is widely used in immunological research to dissect the early steps of immune responses or pathophysiological pathways. It is also growing to be of clinical relevancy to immuno-monitoring and evaluation of the disease status of patients. The techniques currently used for "absolute quantification" of cytokine mRNA are based on a DNA standard curve and do not take into account the critical impact of RT efficiency.

**Results:**

To overcome this pitfall, we designed a strategy using external RNA as standard in the RT-PCR. Use of synthetic RNA standards, by comparison with the corresponding DNA standard, showed significant variations in the yield of retro-transcription depending the target amplified and the experiment. We then developed primers to be used under one single experimental condition for the specific amplification of human IL-1β, IL-4, IL-10, IL-12p40, IL-13, IL-15, IL-18, IFN-γ, MIF, TGF-β1 and TNF-α mRNA. We showed that the beta-2 microglobulin (β2-MG) gene was suitable for data normalisation since the level of β2-MG transcripts in naïve PBMC varied less than 5 times between individuals and was not affected by LPS or PHA stimulation. The technique, we named CyProQuant-PCR (Cytokine Profiling Quantitative PCR) was validated using a kinetic measurement of cytokine transcripts under *in vitro *stimulation of human PBMC by lipopolysaccharide (LPS) or *Staphylococcus aureus *strain Cowan (SAC). Results obtained show that CyProQuant-PCR is powerful enough to precociously detect slight cytokine induction. Finally, having demonstrated the reproducibility of the method, it was applied to malaria patients and asymptomatic controls for the quantification of TGF-β1 transcripts and showed an increased capacity of cells from malaria patients to accumulate TGF-β1 mRNA in response to LPS.

**Conclusion:**

The real-time RT-PCR technique based on a RNA standard curve, CyProQuant-PCR, outlined here, allows for a genuine absolute quantification and a simultaneous analysis of a large panel of human cytokine mRNA. It represents a potent and attractive tool for immunomonitoring, lending itself readily to automation and with a high throughput. This opens the possibility of an easy and reliable cytokine profiling for clinical applications.

## Background

Cytokines are a family of low-molecular weight proteins secreted by various cell types, with pleiotropic functions and constitute a tightly regulated network that plays a central role in the immune system. Cytokines, classified into different groups such as interleukins (IL), interferons (IFN), colony-stimulating factors (CSF), tumour necrosis factors (TNF), tumour growth factors (TGF) and chemokines are implicated in the differentiation, proliferation, migration and effector functions of immune cells. Interacting one with the others, they have polarizing effects on the target cells and are pivotal in tuning immune responses [1]. Therefore, it is rather the make-up of cytokines milieu that influences the immune response rather than the action of a single cytokine. Numerous studies indicate that the clinical and/or immunological status depends on the balance between pro-inflammatory cytokines and their regulatory counterparts [2]. Thus, cytokine profiling should be achieved through analysis of simultaneous quantification of a pattern of cytokines including pro and anti-inflammatory cytokines [2,3]. Moreover, recent reports have highlighted the need for clinical immuno-monitoring of patients to adapt treatment or prevent relapses [4-6]. Thus, analysis of the cytokine pattern is central not only in the definition of the immunological status of patients but also in the study of the pathophysiological pathways as well as the cellular subpopulations involved [7,8].

Cytokines are often produced locally so that the concentration of circulating cytokines in the plasma is usually low. Their half-life and turnover may vary complicating the delineation of informative cytokine profiles. Although transcription of messenger RNA is not strictly correlated to protein secretion and activity, detection of cytokine RNA by real time PCR is now considered a reference technique for analysis of small-size samples with high sensitivity [9]. It can be used on its own or to validate and complement information obtained with other techniques such as micro-arrays [10,11].

The already available techniques, which offer a so-called "absolute quantification" of the target cytokine mRNA, achieve quantification by reference to an external standard curve based on serial dilutions of a known amount of the corresponding cDNA [12]. Moreover, to allow for comparison between experiments, data are normalized by reference to an internal standard, which is an endogenous gene for which the number of copy per cell is supposed constant under different experimental conditions [13,14]. The term of "absolute" quantification is not completely appropriate since these techniques neither control for the variable efficiency of the RT step nor take it into account in their measurements [15,16].

In the present study, we first show that the efficiency of the RT step depends on the target mRNA and on the experiments and that these variations have critical impact on the reliability of mRNA quantification. To overcome this, we describe here CyProQuant-PCR, a new technique for absolute measurement of cytokine mRNA based on an external RNA standard curve. Primer pairs have been designed for allowing amplification of a set of cytokine mRNA using the same conditions both in terms of thermocycling parameters and master mix components, a prerequisite for multiple cytokine mRNA measurements with high throughput.

In the present paper, we describe i) the construction of the synthetic RNA standard, ii) the primer pairs specific for the following human cytokines IL-1β, IL-4, IL-10, IL-12p40, IL-13, IL-15, IL-18, IFN-γ, and for the tyrosine 3-monooxygenase/tryptophan 5-monooxygenase activation protein, zeta polypeptide (YWHAZ), the β2-microglobulin (β2-MG) and the ubiquitin-C (UBC) to be used as internal standards and iii) the conditions for efficient real time amplification of multiple cytokine specific mRNA. The technique was validated using *in vitro *stimulated PBMC and its intra and inter-experimental variability were assessed. Finally, CyProQuant-PCR was used to quantify TGF-β1 transcripts in small blood samples from children with acute *Plasmodium falciparum *malaria.

## Results

### Primer design and validation

**Figure 1:**
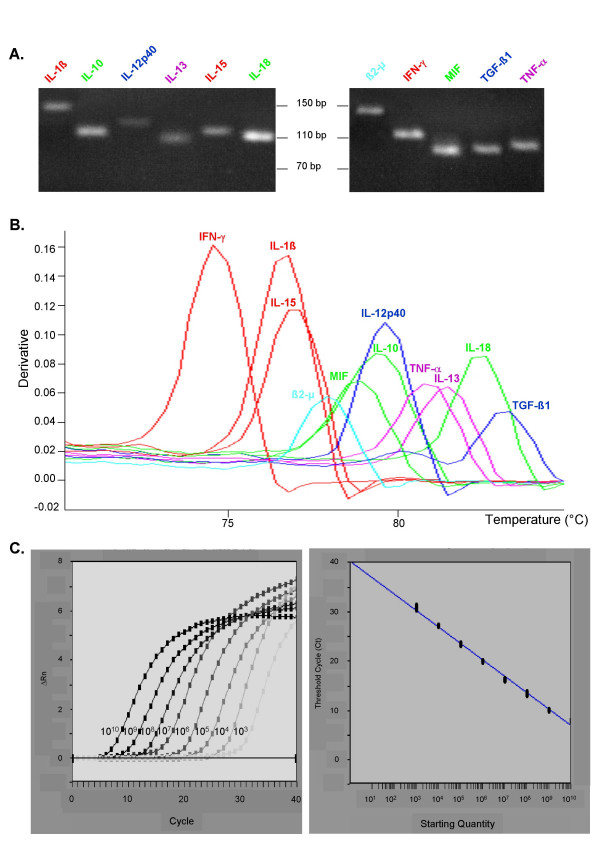
**Primer validation. A. **Agarose gel electrophoresis of several CyProQuant-PCR products generated by amplification of a pool of cDNA from PBMC stimulated *in vitro *by LPS or PHA. Scale is shown in base pairs (bp). **B. **Dissociation curve analysis of these CyProQuant-PCR products. Negative derivative of the fluorescence is plotted against temperature. The single peak shows that SYBR Green fluorescence detects only the specific CyProQuant-PCR product. **C. **Amplification plots and standard curve resulting from the amplification of a range of β2-MG external RNA standard using CyProQuant-PCR.

Primer pairs were designed from published genomic sequences using Primer Express software (Applied Biosystems), except for the UBC and the YWHAZ genes for which the primers had already been described [17]. When possible, the following criteria were applied. The percent of G+C content was kept in the 20–80% range and runs of an identical nucleotide were avoided. The five nucleotides at the 3' end had no more than two G and/or C bases and the melting temperature was kept between 58 and 60°C. Among the primers proposed by Primer Express software, we selected forward and reverse primers amplifying a product spanning one intron and not leading to the amplification of pseudogenes or other related genes to secure primer specificity for target cDNA. The specificity of the amplification was assessed for each gene by electrophoresis and dissociation curve analysis as shown in Figure 1A and 1B. The absence of any contaminating bands corresponding to genomic DNA amplification on agarose gel as well as the presence of a unique peak on the dissociation curve validated the specificity of the primer pair for the target cDNA. PCR products were systematically sequenced after cloning and showed more than 98% identity with the expected sequence (data not shown).

Primer concentrations were optimised to determine the minimum primer concentrations giving the lowest threshold cycle (C_T_) and the maximum signal-to-noise fluorescence ratio (ΔR_n_) while minimising non-specific amplification (data not shown). Among the final concentrations of 50, 300 and 900 nM tested for each primer, the optimal final concentration was set at 900 nM for every primer. The primer pairs are shown in Table I.

### Standard curve design

#### Generation of the RNA standard curve

To generate the standard RNA corresponding to each target sequence, gene specific primers were fused in their 5' end to the RNA polymerase T7 promoter sequence. The PCR performed with these modified primers pairs lead to a larger PCR product with the T7 promoter sequences upstream and downstream from the specific amplicon. The *in vitro *transcription gave a synthetic RNA, which was assessed for its integrity and clonality by electrophoresis (data not shown). The molecular mass of each RNA standard was calculated on the basis of its sequence and solutions ranging from 10^1 ^to 10^12 ^copies of standard RNA were made.

These serial diluted solutions were reverse transcribed and the cDNA amplified in duplicate to generate a standard curve by plotting the threshold cycle (C_T_) against the logarithmic value of the starting RNA copy number for each dilution. Figure 1C shows an example of these curves for β2-MG. Every curve generated a dynamic range of a least 6 orders of magnitude. This allowed for a reliable and reproducible quantification of cellular mRNA sample.

#### Comparison between CyProQuant-PCR and DNA standard based approach

CyProQuant-PCR was compared to the classical approach based on a DNA standard curve for the quantification of TNF-α, IL-1β and IFN-γ transcripts. DNA templates for RNA transcription of TNF-α, IL-1β and IFN-γ were used to generate a range of concentration from 10 to 10^12 ^copies/μl. We thus disposed of DNA and RNA ranges stemming from the same sequence. In parallel, a range of cDNA was also generated from the RNA standards. Ranges of RNA standard were reverse transcribed and then amplified in parallel to the cDNA and DNA ranges by real time PCR under the same conditions (same mix, same final volume, same PCR plate). The slope of the standard curves generated by these three ranges as well as the corresponding efficiency are shown in Table 2. The data show that the RT-PCR efficiencies from CyProQuant-PCR are lower than the PCR efficiencies from cDNA or DNA ranges for the three genes. Since the PCR efficiencies of the cDNA and DNA ranges are similar for each gene, this discrepancy is due to the RT efficiency, which is lower than 100% (from 75% for IFN-γ to 98% for TNF-α). In addition, data show that RT efficiency displays intergenic variation with a lower efficiency for IFN-γ compared to the two other cytokines (Table 2).

In order to show that these intergenic differences were not due to interassay variation, we compared amplification efficiencies of TNF-α, β2-MG, MIF and UBC from i) a single cellular RNA sample, ii) a pooled RNA sample containing standard RNA for each of the targets and iii) a pooled DNA sample corresponding to the cDNA obtained from the pooled RNA sample containing standard RNA for each of the targets. Results obtained are summarised in Table 3 and show that RNA amplification efficiencies vary from one gene to the other but are not affected by the molecular context since they do not differ between the single cellular RNA sample and the pooled RNA sample. Moreover, since the amplification efficiencies of the pooled DNA sample have been normalised to 100%, the difference of amplification efficiency between the RNA samples and the pooled DNA corresponds to differences in RT efficiency.

Altogether, these data demonstrate that RT efficiency displays intergenic variations that are not due to interassay differences.

#### Impact of RT efficiency variability on transcripts quantification reliability

RT efficiency has critical impact on transcript quantification as shown in Table 4. Indeed, if we compare CyProQuant-PCR or DNA standard based approach for quantification of a known number of TNF-α, IL-1β and IFN-γ transcripts, results show that DNA standard based approach underestimates of around two logs the number of transcripts. Moreover, this underestimation varies according to the transcript studied and the experiment. CyProQuant-PCR is thus more precise and more reliable than the classically used DNA-based approaches.

Taken together, these results support the use of RNA as external standard for reliable and reproducible quantification of transcripts.

#### RT-PCR efficiency is similar for sample and RNA standard

Since RT-PCR efficiency varies, absolute mRNA quantification can only be reliably obtained if the external RNA standard and the cellular RNA are retro-transcribed and amplified with the same efficiency. This was secured by comparing the standard curves obtained after amplification of a range of 10X serial dilution of cellular RNA and β2-MG external RNA standard. The slopes obtained were of -3,307 and -3,305 for the cellular RNA and for the external RNA standard, respectively. This corresponds to efficiencies of 100,6% and 100,7% respectively (data not shown).

#### Endogenous standard, normalization and reliability of the technique

The choice of a stable expressed endogenous gene to be used as an internal standard is a prerequisite for accurate RT-PCR expression profiling. This has to be adapted to the clinical situation and the tissue of origin of the samples. Since our purpose was to establish a technique to be used with peripheral blood leukocytes, we tested three genes reported in stable amounts in leukocytes: the β2-MG, the UBC and the YWHAZ [17]. We compared the stability of the amount of transcript under different conditions of activation. Total cellular RNA was extracted on two different days from the same two aliquots of PBMC stimulated for 3 hours by LPS. Thus, reverse transcription was realised on RNA extracted from the same number of cells. The three endogenous gene transcripts were amplified by CyProQuant-PCR in the same plate in duplicate using the same PCR master mix. Table 5 shows the mean of the results obtained for the two extractions. The data show that β2-MG transcripts are stable with less than 12% of variation whereas UBC and YWHAZ transcript levels showed up to 47% variability depending on the *in vitro *conditions. Moreover, based on the approach recently described by Pachot et al. [18], we assessed the inter-individual variability of the β2-MG basal level using whole blood samples from 6 different healthy donors. All CT values were within 19 and 21,86 cycles, which corresponds to a variation of less than 5 times in gene expression for an overall RT-PCR reaction efficiency of 1,81 (slope = -3,867). This is considered as acceptable for a reference gene [18]. β2-MG was thus chosen as an internal standard gene for future experiments. The same conclusions were drawn using PHA (phytohemagglutinin) as a stimulant (data not shown). In addition, these data validate the reliability and reproducibility of our RNA extraction protocol.

### Validation of the CyProQuant-PCR technique

#### Human TNF-a transcript quantification: comparison of CyProQuant-PCR to TaqMan^®^

**Figure 2:**
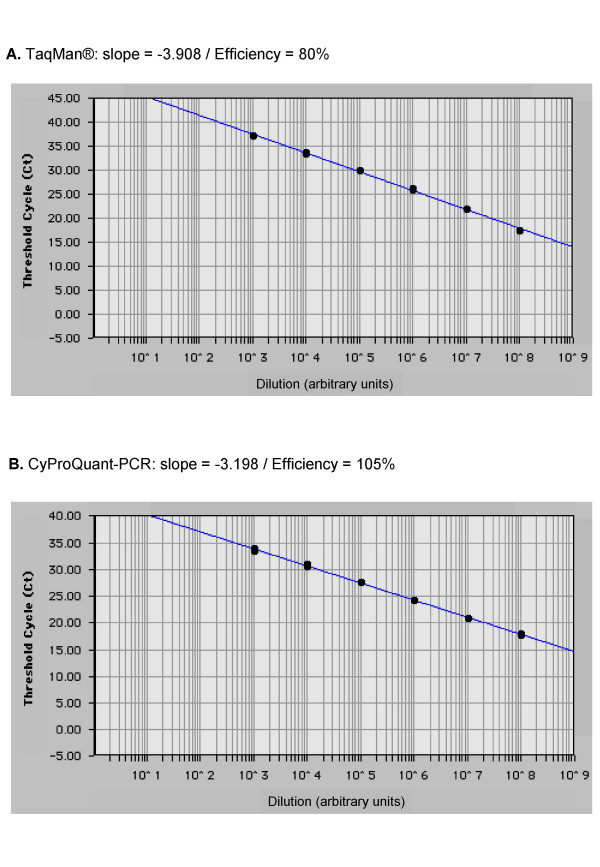
**Comparison to TaqMan^® ^technology. **Total RNA was extracted from isolated monocytes stimulated for 6 hours by LPS, serial-diluted 1:10 and retro-transcribed to generate standard curves by plotting the C_T _against the concentration of this cellular RNA in arbitrary units. TNF-α transcripts were amplified by real time PCR using either (**A**) the TaqMan^® ^commercial kit and the TaqMan^® ^universal PCR master mix or (**B**) our primers and the SYBR Green PCR master mix. Data represent the standard curves obtained for the two techniques, their slopes and the deducted efficiencies.

Since TaqMan^® ^technology developed by Applied Biosystems is considered as the "gold standard", we compared CyProQuant-PCR to TaqMan^® ^for the quantification of TNF-α transcripts in isolated monocytes stimulated for 6 hours with LPS. Total RNA was extracted from 10^6 ^cells and 10X serial dilutions were prepared and reverse transcribed. The resulting cDNA were amplified in duplicate on the same plate in the same thermocycling conditions using either the TaqMan^® ^commercial kit (Applied Biosystems) or CyProQuant-PCR primers with SYBR Green PCR master mix (Applied Biosystems). Figure 2 shows the curves obtained using the two techniques on the same samples. This indicates that CyProQuant-PCR is as good as TaqMan^® ^in terms of sensitivity and even more efficient: 105% (slope -3,198) for CyProQuant-PCR versus 80 % (slope -3,908) for TaqMan^®^.

#### Quantification of TNF-α and MIF by CyProQuant-PCR and ELISA

**Figure 3:**
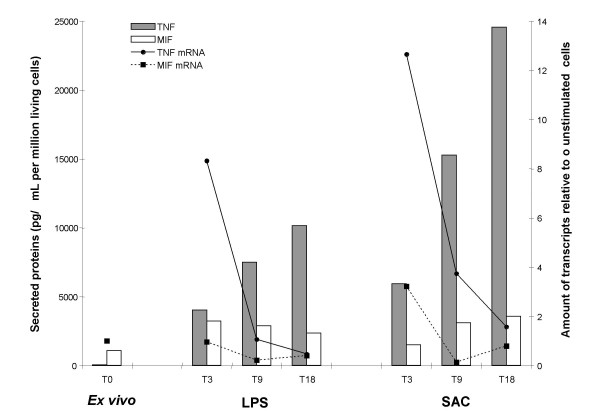
**TNF and MIF transcription and secretion kinetics. **PBMC from 2 donors were stimulated for 3, 9 or 18 hours (T3, T9 and T18 respectively) with LPS or SAC. TNF-α and MIF transcripts were quantified using CyProQuant-PCR and the corresponding secreted proteins were measured by ELISA. Transcript numbers are normalised for one million copies of β2-MG RNA relative to the level of transcripts in un-stimulated cells (fold increase). Secreted proteins are expressed as pg/mL for one million living cells.

To validate our approach, we measured the levels of TNF-α and MIF transcripts and secreted proteins by CyProQuant-PCR and ELISA respectively. PBMC from healthy individuals were stimulated by LPS or SAC for 3, 9 and 18 hours. Figure 3 shows an increase of TNF-α transcripts after 3 hours of stimulation that precedes protein secretion. In contrast, MIF transcripts were constitutively present at the steady state and LPS or SAC stimulation did not significantly modify the level of transcripts although it induces the release of substantial amounts of MIF proteins. This is in agreement with reports showing that MIF exists within cells under homeostasis both as a preformed protein ready to be secreted and as a messenger RNA, which can then be rapidly translated in the absence of induced transcription [19].

#### Early cytokine profiling of PBMC from healthy donors stimulated with LPS or SAC

**Figure 4:**
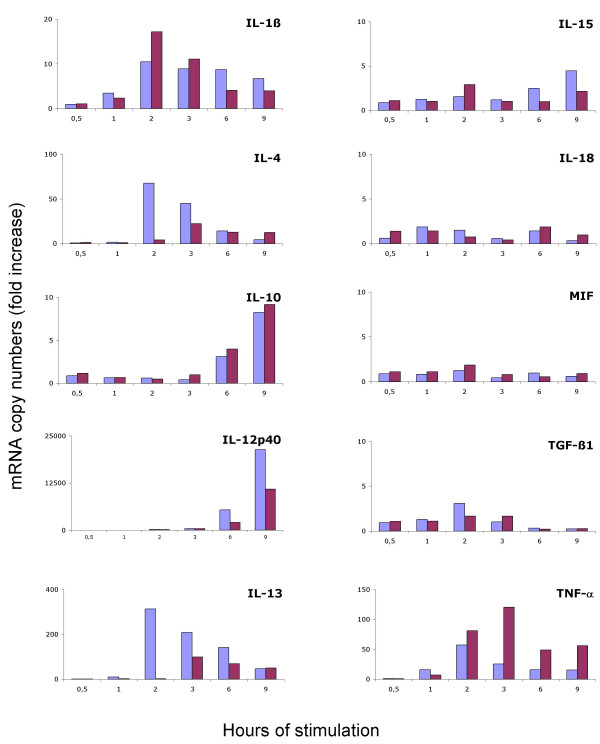
**Early cytokine production kinetics by PBMC stimulated in vitro with LPS or SAC. **Total cellular RNA was extracted from PBMC from 2 donors stimulated for 0.5, 1, 2, 3, 6 or 9 hours with LPS (blue bars) or SAC (red bars). Cytokine transcripts were quantified using CyProQuant-PCR. The results are shown for one donor and were expressed in mRNA copy numbers calculated relative to un-stimulated cells, after normalisation against β2-MG (fold increase).

To validate the panel of primers designed for CyProQuant-PCR, we assessed the early cytokine response as well as the kinetics of expression for PBMC from two healthy donors under stimulation with LPS or SAC. Figure 4 illustrates CyProQuant-PCR detection of IL-1β, IL-4, IL-10, IL-12p40, IL-13, IL-15, IL-18, MIF, TGF-β1 and TNF-α transcripts for one donor. No significant increase in IL-15, IL-18, MIF or TGF-β1 transcripts was detected. Both stimulants induce a similar kinetics of transcript accumulation for IL-1β and IL-10. In contrast, IL-4 and IL-13 transcripts peaked very early (2 hours) under LPS stimulation but not under SAC stimulation. IL-12p40 transcripts also accumulated earlier under LPS stimulation compared to SAC but was detected later on. The amount of TNF-α transcript was sustained under SAC stimulation compared to LPS. Detection of the corresponding proteins for TNF-α, IL-10 and IL-12p40 confirmed the mRNA profile observed (data not shown). The amplitude of the response differed somewhat between the two donors but the kinetics obtained for each cytokine were similar (data not shown).

#### Application: Use of CyProQuant-PCR to quantify TGF-β1 transcripts in malaria patients

**Figure 5:**
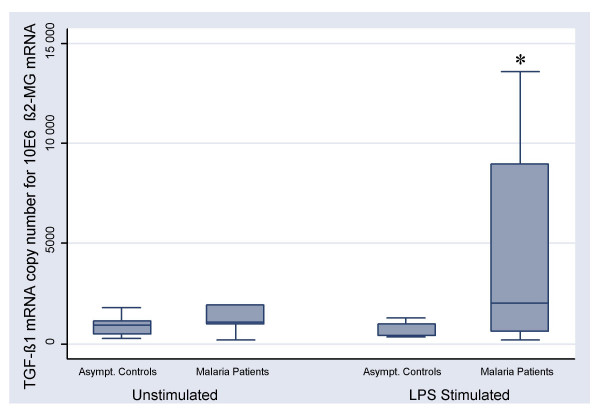
**TGF-β1 transcripts quantification in malaria patients and asymptomatic controls **TGF-β1 transcripts were quantified without stimulation and after 22 hours of LPS stimulation of PBMC harvested from asymptomatic controls (n = 5) and from patients suffering from acute malaria (n = 20). Results are normalised for one million copies of β2-microglobulin. Whiskers indicate data range, boxes extend from the 25^th ^to the 75^th ^percentile and the horizontal lines show the median. Asterisk indicates significant higher value in malaria patients compared to asymptomatic controls (p = 0.036) in the capacity of response to LPS.

Before the application to clinical samples, the experimental reproducibility of CyProQuant-PCR was evaluated using a range of TGF-β1 and β2-MG RNA standards. Inter-experimental variability was assessed from eight independent experiments. The coefficients of variation were satisfactory whatever the starting copy number of RNA standard, ranging from 0,39 to 1,07% and from 0,91 to 1,2% for TGF-β1 and β2-MG respectively (Table 6). TGF-β1 transcripts were then quantified after LPS stimulation of PBMC from malaria patients and asymptomatic controls. Figure 5 shows a significant difference in the amount of TGF-β1 transcripts between patients and asymptomatic controls only after LPS stimulation (p = 0,036).

## Discussion

In this paper we have described a new technique, CyProQuant-PCR, for absolute quantitative profiling of human cytokine mRNA using real time RT-PCR. Although real time PCR is now becoming a popular technique, it still requires improvement for proper quantification. All the techniques for absolute quantification available so far use a DNA standard curve assuming that RT efficiency is constant and approaches 100% [12]. We show here that RT is not 100% efficient, and more importantly, that its efficiency changes from one gene to another and from one experiment to another. We thus designed primers and standard RNA to be used in such a way that all cytokine mRNA of interest as well as three housekeeping genes could be amplified using the same conditions (thermocycling parameters and buffer). This technique is rapid, reliable and reproducible. When compared to the TaqMan^® ^commercial kit, which represents the "gold standard", CyProQuant-PCR was as sensitive but less expensive and more flexible. Indeed, for a given gene, the design of the probe might be quite difficult and, with judicious selection of primer pairs, comparable sensitivity can be achieved with the use of SYBR Green [20].

A major reason for not using an RNA standard curve is its poor stability due to its sensitivity to RNase degradation. In our hands, we did not find any detectable degradation when keeping the standard in concentrated aliquots (stock solution at 1000 μg/mL) in RNase-free water at minus 80°C and avoiding freeze-thawing cycles. However, for a greater stability, we incorporated modified dNTP, 2'-Fluoro-dCTP and 2'-Fluoro-dUTP, which have been reported to decrease the sensitivity of the *in vitro *transcribed RNA to specific RNases [21,22], and tested the effect of this incorporation on the RT-PCR efficiency. We did not find significant difference in the efficiencies (100% for the non-modified IL-4 standard versus 104% for the 2'-Fluoro-standard) (data not shown).

The methodology described here was easily and successfully applied to the quantification of several cytokine genes. The quantification is reliable on 7 to 8 logs with a sensitivity ranging from 1000 to 100 copies depending the cytokine. It was first used to determine the magnitude and the kinetics of early induction of cytokines mRNA upon PBMC stimulation using bacterial derived materials. This showed that CyProQuant-PCR is powerful enough to detect early on modest cytokine induction such as IL-4. Such a tool is useful to decipher the kinetics of cytokine response involved in physiopathological pathways but also as a read-out to measure minor immune responses to specific antigens [23].

We finally applied CyProQuant-PCR to measure the level of TGF-β1 transcripts in asymptomatic controls and malaria patients after LPS stimulation. Interestingly, we observed that cells from malaria patients have a significant higher capacity to respond to LPS compared to controls. The increased TGF-β1 transcript accumulation by patients' cells, suggests that an inadequate production of TGF-β1 might play a role in malaria pathogenesis as already proposed [24]. Further work is needed to elaborate on this finding. This first study provided the proof that CyProQuant-PCR is readily applicable to small clinical samples from paediatric cases. This opens the possibility to further quantify the cytokine imbalance associated with malaria pathogenesis and generate a disease cytokine signature, a prerequisite for novel therapeutic interventions targeting cytokine gene expression. Areas of application include both infectious and non-infectious diseases, as well as chronic inflammatory diseases such as rheumatoid arthritis or sarcoidosis and acute diseases such as sepsis or malaria. Beside its importance in patient immuno-monitoring, cytokine profiling is also a major tool to study specific immune response against antigens for design and testing of immuno-modulatory drugs or vaccines.

## Conclusion

In conclusion, we provide here CyProQuant-PCR, a simple technique for genuine absolute quantification of cytokine mRNA using SYBR Green^® ^which is as sensitive as the TaqMan^® ^technique. Because the parameters of amplification are identical for all the cytokines developed, CyProQuant-PCR is readily automatable notably for 384-well plates and might allow multiple cytokine profiling of samples of very limited size at a relatively high throughput. CyProQuant-PCR opens the possibility to use cytokine mRNA measurements in clinical studies not only for an increased knowledge but also to help clinicians in patients' stratification and treatment decision.

## Methods

### Healthy human PBMC: isolation and *in vitro *stimulation

Blood was collected from healthy donors at the French Blood Bank. Peripheral blood mononuclear cells (PBMC) were isolated by density separation over Ficoll Hypaque and washed two times in RPMI 1640 (Gibco BRL, Invitrogen, Cergy Pontoise, France). Cells were re-suspended in RPMI 1640 (Gibco BRL, Invitrogen, Cergy Pontoise, France) supplemented with 2 mM glutamine (Gibco BRL, Invitrogen, Cergy Pontoise, France) and 10% AB^+ ^human serum (French Blood Bank) at 2.10^6 ^cells/mL and either used directly for RNA extraction or cultured in duplicate with or without LPS (10 ng/mL, *E. Coli *O111: B7, Sigma, L'Isle d'Abeau Chesnes, France) or SAC (0,0075%, PanSorbine Cells, Calbiochem, La Jolla, CA, USA). After incubation, cells were washed with PBS and re-suspended in RNA-PLUS (Q-Biogene, Illkirch, France) for RNA isolation.

### Malaria patients and asymptomatic controls

Twenty children admitted during the high malaria transmission season of 2001 to the emergency room at the Department of Child Health, Korle-Bu Teaching Hospital, Ghana were included. Five asymptomatic controls matched to patients for age, residence location and time of sample collection were enrolled. The general inclusion and exclusion criteria were as described by Kurtzhals *et al. *[25]. Parents or guardians signed informed consent forms. The study received ethical clearance from The Ethics and Protocol Review Committee at the university of Ghana Medical School and the Ministry of Health. Total cellular RNA was extracted from PBMC recovered from 500 μL of blood following supplier's instructions (RNA PLUS, Q-Biogene, Illkirch, France) after 22 hours of incubation at 37°C, 5% CO2 with or without LPS (10 ng/mL, *E. Coli *O111: B7, Sigma, L'Isle d'Abeau Chesnes, France).

### RNA isolation

RNA was extracted following supplier's instructions, re-suspended in 60 μL of RNase-free water (Ambion, Huntingdon, UK) and quantified spectrophotometrically at 260 nm.

### Primers

Oligonucleotide primers were synthesized at Eurogentec (Saraing, Belgium). To validate primers, a pool of cDNA from healthy human PBMC stimulated for 6 and 12 hours with LPS (10 ng/mL, *E. Coli *O111: B7, Sigma, L'Isle d'Abeau Chesnes, France) and PHA-L (10 μg/mL, Sigma-Aldrich, Lyon, France) was used. Analysis of the amplicons was assessed by 4% agarose gel electrophoresis and dissociation curve studies using Dissociation Curve Software (Applied Biosystems, Foster City, CA, USA). PCR products were cloned into pCR2.1 vector using Original TA cloning kit (InVitrogen, Cergy Pontoise, France) and sequenced (Genome Express, Meylan, France).

### Construction of the external DNA and RNA standards

PCR products generated by each primer pairs were column-purified (Nucleospin, Macherey-Nagel, Hoerdt, France) and quantified spectrophotometrically at 260 nm. The molecular weight of the standard DNA was calculated by N*487-[(N-1)*175] were N is the number of bases composing the standard DNA. Stock solution of 10^12 ^copies of standard DNA /3,85 μL were made in Tris-EDTA buffer (Ambion, Huntingdon, UK), split in single-use aliquots and stored at -80°C in safe-lock tubes. External DNA standard range was made extemporaneously by 1:10 serial dilutions in water.

Gene specific primers were fused on their 5' end to the sequence of the RNA polymerase T7 promoter to generate modified primers. These primers were used to amplify a gene specific PCR product flanked by transcription initiation sites. Five hundred nanograms of this construct were *in vitro *transcribed (MegaShortScript, Ambion, Huntingdon, UK). The standard RNA generated was purified (MegaClear, Ambion, Huntingdon, UK) and loaded on a 4% agarose gel for electrophoresis. The concentration of the standard RNA was determined spectrophotometrically at 260 nm. The molecular weight of the transcript was calculated by N*500-[(N-1)*175] were N is the number of bases composing the standard RNA. Stock solution of 10^12 ^copies of standard RNA /3,85 μL were made in RNA storage solution (Ambion, Huntingdon, UK), split in single-use aliquots and stored at -80°C in safe-lock tubes. External RNA standard range was made extemporaneously by 1:10 serial dilutions in water.

### Reverse transcription

For CyProQuant-PCR assays, 100 ng of total cellular RNA from PBMC and serial dilution of external RNA standard were reverse transcribed simultaneously in a parallel procedure using Reverse Transcription TaqMan reagents (Applied Biosystems, Foster City, CA, USA) on a MasterCycler Gradient (Eppendorf, Le Pecq, France). The final volumes were set at 100 μL for the cellular RNA samples and 50 μL for the external RNA standard range. The thermocycling parameters were as follows: 25°C, 10 min.; 48°C, 60 min. and 95°C, 5 min. cDNA were immediately used for PCR amplification.

### Real-time RT-PCR and quantification of transcripts

Reverse-transcribed standard RNA and cellular RNA were amplified simultaneously on the same PCR plate on an ABI Prism 7700 (Applied Biosystems, Foster City, CA, USA). An aliquot of 5 μL of the RT reaction was amplified in duplicate in a final volume of 30 μL of SYBR Green PCR Master mix (Applied Biosystems, Foster City, CA, USA). Thermocycling conditions were 50°C for 2 min., 95°C for 10 min. and 40 cycles of [95°C/15 sec.; 60°C, 1 min]. The sample target RNA copy numbers were calculated using SDS 1.9 Software (Applied Biosystems, Foster City, CA, USA). The baseline fluorescence was set manually to correct for differences in initial cDNA concentration and the threshold was positioned at a fluorescence level that was 10 times higher than the background signal. Target mRNA copy numbers in cellular samples were calculated based on a standard curve generated by SDS 1.9 Software (Applied Biosystems, Foster City, CA, USA) by plotting cycles at threshold (C_T_) against the logarithmic values of the starting RNA standard copy number.

### ELISA tests

TNF-α and MIF secreted proteins were quantified by sandwich ELISA following supplier's instructions (Bio-Source, Clinisciences, Montrouge, France and R&D, Lille, France respectively). Results are expressed as pg/mL for one million living cells.

### Statistical analysis

Tests for significance were done using Stata software (Stata Corporation, College Station, Texas, 77845 USA) by Kruskal-Wallis rank test.

### Patent application

Results disclosed in this manuscript have been protected in French patent application FR0408645.

## Authors' contributions

PB developed the entire technique. IV did the *in vitro *stimulation experiments. DJ gave technical assistance. SL helped in RNA extraction of malaria samples. JCB introduced PB to molecular biology techniques and provided critical advices. BDA designed and conducted the study that yielded the malaria samples. OMP revised the manuscript and supported the work. CB conceived the strategy and coordinated the study. PB and CB drafted the manuscript. All authors read and approved the final manuscript.

## Figures and Tables

**Table 1:** Primer sequences used for Cyproquant assays Table shows position of amplification product within cDNA sequence (upper line) and within genomic sequence (lower line). GenBank accession numbers are indicated.

**Gene name**	**5'-3' primer sequence**		**Position cDNA-gene**	**Accesion Number**
IL-1β	FW	CCTGTCCTGCGTGTTGAAAGA	639–788	NM_000576
	RW	GGGAACTGGGCAGACTCAAA	Int. Span.	M15840
IL-4	FW	AACAGCCTCACAGAGCAGAAGAC	177–278	BC070123
	RW	GCCCTGCAGAAGGTTTCCTT	Int. Span.	AF395008
IL-10	FW	GCTGGAGGACTTTAAGGGTTACCT	210–318	AY029171
	RW	CTTGATGTCTGGGTCTTGGTTCT	Int. Span.	AF418271
IL-12p40	FW	CATGGTGGATGCCGTTCA	636–767	AF180563
	RW	ACCTCCACCTGCCGAGAAT	Int. Span.	AY008847
IL-13	FW	ACAGCCCTCAGGGAGCTCAT	135–231	NM_002188
	RW	TCAGGTTGATGCTCCATACCAT	Int. Span.	AF377331
IL-15	FW	CCATCCAGTGCTACTTGTGTTTACTT	351–466	U14407
	RW	CCAGTTGGCTTCTGTTTTAGGAA	Int. Span.	X91233
IL-18	FW	GACGCATGCCCTCAATCC	966–1071	NM_001562
	RW	CTAGAGCGCAATGGTGCAATC	Exon	E17138
IFN-γ	FW	GTTTTGGGTTCTCTTGGCTGTTA	151–263	NM_000619
	RW	AAAAGAGTTCCATTATCCGCTACATC	Int. Span.	AF375790
MIF	FW	GCCCGGACAGGGTCTACA	322–399	M25639
	RW	CTTAGGCGAAGGTGGAGTTGTT	Int. Span.	L19686
TGF-β1	FW	CAAGGGCTACCATGCCAACT	1786–1869	NM_000660
	RW	AGGGCCAGGACCTTGCTG	Int. Span.	X05839
TNF-α	FW	CCCCAGGGACCTCTCTCTAATC	358–455	NM_000594
	RW	GGTTTGCTACAACATGGGCTACA	Int. Span.	AB088112
β2-MG	FW	AATTGAAAAAGTGGAGCATTCAGA	255–389	NM_004048
	RW	GGCTGTGACAAAGTCACATGGTT	Exon	M17987
UBC	FW	ATTTGGGTCGCGGTTCTTG	20–152	BC039193
	RW	TGCCTTGACATTCTCGATGGT	Int. Span.	D63791
YWHAZ	FW	ACTTTTGGTACATTGTGGCTTCAA	1090–1183	NM_003406
	RW	CCGCCAGGACAAACCAGTAT	Exon	NC_000008

Note: FW = Forward primer; RW = Reverse primer; Int. Span. = Intron spanning, i.e. Primers derived from different exons; Exon = both primers derived from the same exon. β2-MG = β2-microglobulin. UBC = Ubiquitin C. YWHAZ = tyrosine 3-monooxygenase tryptophan 5-monooxygenase activation protein, zeta polypeptide.

**Table 2:** Comparison between CyProQuant-PCR and DNA standard-based approaches Efficiencies (E) are deducted from the slopes (S) of the standard curves based on E = 100*(10^-1/S ^- 1). The deducted RT efficiency from the CyProQuant-PCR is calculated by dividing the RT-PCR efficiency from the RNA range (E_RT-PCR_) by the PCR efficiency from the cDNA range (E_PCR_).

Target gene	**CyProQuant-PCR**					**DNA standard-based PCR**	
	
	RNA range		cDNA range		Deducted RT efficiency	DNA range	
			
	Slope	E_RT-PCR_	Slope	E_PCR_		Slope	E_PCR_
TNF-α	-3.610	89	-3.556	91	98	-3.503	93
IL-1β	-3.479	94	-3.197	105	89	-3.067	112
IFN-γ	-3.843	82	-3.116	109	75	-2.944	119

**Table 3:** Intergenic difference of RT efficiencies is not due to interassay variation. Serial 1:10 dilutions of a single cellular RNA sample, of a pooled RNA sample and of a pooled DNA sample were used to generate standard curves for different targets (TNF-α, β2-MG, MIF and UBC). Slopes were indicative of amplification efficiencies, which were normalised to 100% for the pooled DNA sample for easier comparison to amplification efficiencies of the two other samples.

	**Single cellular RNA sample**	**Pooled RNA sample**	**Pooled DNA sample**
**TNF-α**	95	95	100
**β2-MG**	90	90	100
**MIF**	88	88	100
**UBC**	81	80	100

**Table 4:** RNA and DNA standard-based quantification of TNF-a, IL-1β and IFN-γ transcripts Fifty million copies of TNF-α, IL-1β and IFN-γ standard RNA were amplified in parallel of a range of RNA standard (CyProQuant-PCR) and DNA standard. Copy numbers were directly deduced from the cycle at threshold. *For TNF-α, the experiment was repeated at two days interval.

Target gene	Actual copy number	Calculated copy number	
		
		CyProQuant-PCR	DNA standard approach
TNF-α*	5 10^7^	5.2 10^7^	7.7 10^5^
	5 10^7^	5.0 10^7^	3.6 10^5^
IL-1β	5 10^7^	4.3 10^7^	6.7 10^5^
IFN-γ	5 10^7^	5.5 10^7^	7.8 10^5^

**Table 5:** Expression stability of endogenous standard genes under non-normalised conditions After 3 hours of stimulation with LPS, PBMC from healthy donors were split in 2 identical aliquots and total cellular RNA was extracted at 2 days interval. The same volume of RNA was reverse transcribed and β2-MG, UBC and YWHAZ transcripts were amplified by real time PCR using CyProQuant-PCR technique. Results show the mean values obtained for the 2 extractions in arbitrary units.

Stimulation conditions	Molecules detected (arbitrary units)	
	
	T0	LPS 3 h
β2-MG	1.24 10^7^	1.48 10^7^
	(1.00 10^7 ^– 1.49 10^7^)	(1.17 10^7 ^– 1.79 10^7^)
UBC	2.04 10^5^	2.39 10^5^
	(0.62 10^5 ^– 3.46 10^5^)	(1.51 10^5 ^– 3.28 10^5^)
YWHAZ	1.89 10^5^	2.80 10^5^
	(0.78 10^5 ^– 3.01 10^5^)	(2.25 10^5 ^– 3.34 10^5^)

**Table 6:** Inter-experimental reproducibility of the quantification of a range of TGF-β1 and β2-MG RNA standards A set of TGF-β1 and β2-MG RNA standards ranging from 10^3 ^to 10^10 ^copies was amplified by RT-PCR. Coefficients of inter-experimental variation were determined from eight different experiments and calculated for threshold cycles.

Absolute copy number	Coefficients of inter-experimental variations (%)	
	
	TGF-β1	β2-MG
1.00E+03	1.04	1.20
1.00E+04	1.07	1.19
1.00E+05	0.94	1.13
1.00E+06	0.90	1.08
1.00E+07	0.79	0.95
1.00E+08	0.71	0.91
1.00E+09	0.54	0.93
1.00E+10	0.39	1.18
**Average**	**0.80**	**1.07**
